# Kaempferol Suppresses the Activation of Mast Cells by Modulating the Expression of FcεRI and SHIP1

**DOI:** 10.3390/ijms24065997

**Published:** 2023-03-22

**Authors:** Kazuki Nagata, Sanae Araumi, Daisuke Ando, Naoto Ito, Miki Ando, Yuki Ikeda, Miki Takahashi, Sakura Noguchi, Yayoi Yasuda, Nobuhiro Nakano, Tomoaki Ando, Mutsuko Hara, Takuya Yashiro, Masakazu Hachisu, Chiharu Nishiyama

**Affiliations:** 1 Department of Biological Science and Technology, Faculty of Advanced Engineering, Tokyo University of Science, 6-3-1 Niijuku, Katsushika-ku, Tokyo 125-8585, Japan; 2 Atopy (Allergy) Research Center, Juntendo University Graduate School of Medicine, 2-1-1 Hongo Bunkyo-ku, Tokyo 113-8421, Japan

**Keywords:** bone marrow-derived mast cells, FcεRI, IgE, IL-33, kaempferol, LPS, NRF2, SHIP1

## Abstract

In the present study, we evaluated the effects of kaempferol on bone marrow-derived mast cells (BMMCs). Kaempferol treatment significantly and dose-dependently inhibited IgE-induced degranulation, and cytokine production of BMMCs under the condition that cell viability was maintained. Kaempferol downregulated the surface expression levels of FcεRI on BMMCs, but the mRNA levels of FcεRIα, β, and γ-chains were not changed by kaempferol treatment. Furthermore, the kaempferol-mediated downregulation of surface FcεRI on BMMCs was still observed when protein synthesis or protein transporter was inhibited. We also found that kaempferol inhibited both LPS- and IL-33-induced IL-6 production from BMMCs, without affecting the expression levels of their receptors, TLR4 and ST2. Although kaempferol treatment increased the protein amount of NF-E2-related factor 2 (NRF2)—a master transcription factor of antioxidant stress—in BMMCs, the inhibition of NRF2 did not alter the suppressive effect of kaempferol on degranulation. Finally, we found that kaempferol treatment increased the levels of mRNA and protein of a phosphatase SHIP1 in BMMCs. The kaempferol-induced upregulation of SHIP1 was also observed in peritoneal MCs. The knockdown of SHIP1 by siRNA significantly enhanced IgE-induced degranulation of BMMCs. A Western blotting analysis showed that IgE-induced phosphorylation of PLCγ was suppressed in kaempferol-treated BMMCs. These results indicate that kaempferol inhibited the IgE-induced activation of BMMCs by downregulating FcεRI and upregulating SHIP1, and the SHIP1 increase is involved in the suppression of various signaling-mediated stimulations of BMMCs, such as those associated with TLR4 and ST2.

## 1. Introduction

Mast cells (MCs) play important roles in allergic diseases, particularly IgE-dependent allergic responses induced by the cross-linking of the cell-type specifically expressed high affinity receptor for IgE, FcεRI, on the surface. The cross-linking of IgE-binding FcεRIs with antigen (Ag) induces the activation of MCs, resulting in the release of various mediators, such as histamine and eicosanoids, and the transactivation of cytokine genes in MCs, which are involved in rapid and late-phase allergic responses.

Polyphenols are the secondary metabolites of plants and belong to the phytochemicals, which are produced in plants to resistant stresses and provide a variety of health benefits for humans and animals. Several polyphenols inhibit the IgE-induced activation of MCs in vitro or ameliorate IgE-dependent allergic responses in vivo [1,2,3,4,5]. Flavonoids are the most vigorously studied polyphenols, including luteolin, nobiletin, quercetin, and kaempferol. Kaempferol, which is abundant in fruits and vegetables and is also found in plant-derived beverages, is reported to inhibit allergic diseases in model mice [6,7,8,9]. Several in vitro studies using cell lines, such as rat basophilic leukemia cell line RBL-2H3, human basophilic cell line KU812F, human MC lines LAD2 and HMC-1, suggested that IgE-induced activation of MCs was suppressed by kaempferol or its related flavonoids, including quercetin [7,8,9,10,11,12]. A valuable study using human umbilical cord blood-derived culture MCs revealed that several flavonols, including kaempferol, inhibited intracellular calcium influx in and proinflammatory mediator release from IgE-stimulated human MCs [13]. However, the molecular mechanisms underlying the inhibited activation of MCs remain unclear.

In the present study, we used bone marrow-derived MC (BMMC) and found that kaempferol suppressed the IgE-induced degranulation and cytokine release of BMMCs. Further analyses using flowcytometry and qPCR showed that the cell surface expression level of FcεRI was downregulated by kaempferol, which was parallel to the suppression levels of the IgE-induced activation of BMMCs and was caused in a transcription-independent manner. In addition, the activation of BMMCs by LPS or IL-33 was significantly inhibited by kaempferol, even though the expression of TLR4 and ST2 was not affected by kaempferol. To reveal intracellular events modulated by kaempferol, we investigated the involvement of NF-E2-related factor 2 (NRF2), which is a master transcription factor of antioxidant stress and is activated by several phytochemicals exerting anti-inflammatory effects [14]. We also evaluated the effects of kaempferol on phosphatases that are negative regulators of signal transduction induced by various stimuli [15], and observed that kaempferol upregulated the expression of a phosphatase, SHIP1 in BMMCs, and peritoneal MCs.

## 2. Results

### 2.1. Kaempferol Suppressed IgE-Mediated Activation of BMMCs

**Figure 1:**
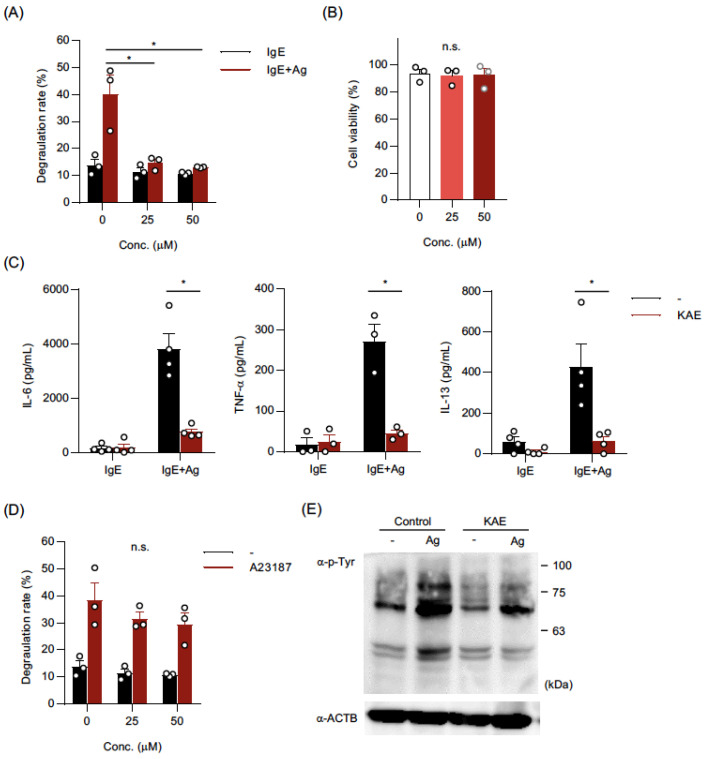
Kaempferol suppressed IgE-mediated activation of BMMCs. BMMCs were pre-incubated in the presence or absence of kaempferol (KAE) (50 μM or indicated concentrations) for 24 h followed by each assay. (**A**) Effects of KAE on the IgE-mediated degranulation of BMMCs. After sensitization with anti-TNP-IgE, BMMCs were incubated in Tyrode’s buffer with or without TNP-BSA, and the supernatant and cells were collected for β-hexosaminidase assay. (**B**) Effects of KAE on the viability of BMMCs. DAPI-stained cells were judged to be dead cells. (**C**) Inhibition of cytokine release from BMMCs by KAE. IgE-sensitized BMMCs were incubated in culture medium with or without TNP-BSA for 3 h and the supernatant was collected. (**D**) Effects of KAE on the degranulation of BMMCs induced by A23187. BMMCs were incubated in Tyrode’s buffer with or without A23187, and the supernatant and cells were collected for β-hexosaminidase assay. (**E**) Western blot analysis of tyrosine-phosphorylated proteins. BMMCs were stimulated with IgE plus TNP-BSA for 10 min. Whole cell lysate containing 10 μg protein was added to each lane. The data shown in (**A**–**D**) represent the mean ± SEM of 3~4 independent experiments, respectively. The data shown in (**E**) represents a typical data of three other independent experiments. Dunnett’s multiple comparison test (**A**,**D**) and two-tailed paired *t*-test (**C**) were used for statistical analyses. *, *p* < 0.05; n.s., not significant. Abbreviation: Conc., concentration; Ag, Antigen; KAE, Kaempferol.

To evaluate the effects of kaempferol on the activation of BMMCs, we incubated BMMCs in the presence of various concentrations of kaempferol and found that the preincubation of BMMCs with 25–50 μM of kaempferol for 24 h before the addition of IgE significantly suppressed IgE-mediated degranulation (Figure 1A) without affecting cell viability (Figure 1B). A 24 h pretreatment with 50 μM kaempferol also drastically inhibited the release of cytokines, including IL-6, TNF-*α*, and IL-13, from IgE-stimulated BMMCs (Figure 1C). In contrast, although the pretreatment with kaempferol tended to reduce A23187-induced degranulation of BMMCs, the effects were not significant (Figure 1D). A Western blot using an anti-phospho-Tyr antibody (Ab) revealed that the IgE-induced increase in phosphorylated Tyr residues in intracellular proteins was suppressed by the kaempferol treatment (Figure 1E).

These results demonstrate that kaempferol effectively suppressed IgE-mediated activation of BMMCs, mainly targeting the signal transductions located between downstream of IgE and upstream of Ca^2+^-signaling.

### 2.2. Kaempferol Reduced the Cell Surface Levels of FcεRI on BMMCs

**Figure 2:**
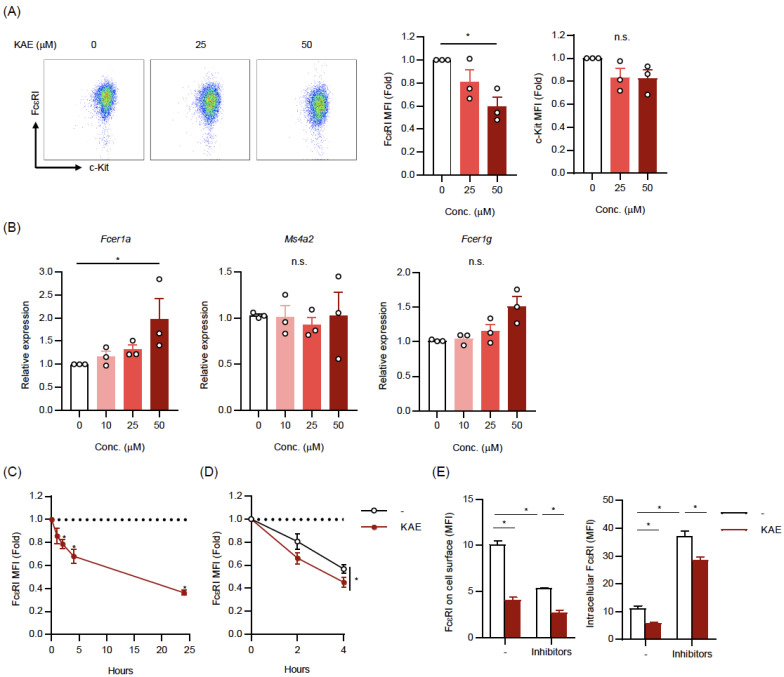
Kaempferol reduced the cell surface levels of FcεRI on BMMCs. (**A**) Cell surface expression levels of FcεRI and c-kit. Typical dot plot profiles (left) and the mean fluorescence intensity (MFI) of FcεRI (middle) and c-kit (right) were obtained in three independent experiments. BMMCs incubated in the presence or absence of the indicated concentrations of KAE for 24 h were stained with anti-FcεRI Ab and anti-c-kit Ab. (**B**) mRNA expression levels of FcεRIα (*Fcer1a*), β (*Ms4a2*), and γ (*Fcer1g*) subunits. BMMCs pretreated with KAE for 24 h were harvested to assess mRNA levels by qPCR (normalized by β-actin). (**C**) Time course of cell surface expression levels of FcεRI following KAE treatment. BMMCs were incubated in the presence of KAE (50 μM) and harvested at indicated time. (**D**) Time course of cell surface expression levels of FcεRI following KAE treatment in the presence of cycloheximide. Cycloheximide-treated BMMCs were incubated in the presence or absence of KAE (50 μM) and harvested at indicated time. (**E**) The cell surface (left) and intracellular (right) expression levels of FcεRI following KAE treatment with or without protein transporter inhibitors. BMMCs were treated with KAE (50 μM) in the presence or absence of Brefeldin A (5 μg/mL) and Monensin (2 μM) for 6 h. After labeling surface FcεRI with FITC-labeled Ab, cells were permeabilized and stained with PE-labeled Ab. The data represent the mean ± SEM of 3~5 independent experiments (**A**–**D**) and the mean ± SD of a typical data of triplicated samples from three independent experiments (**E**). Dunnett’s multiple comparison test (**A**–**C**), two-tailed paired *t*-test (**D**) and Tukey’s multiple comparison test (**E**) were used for statistical analyses. *, *p* < 0.05; n.s., not significant. Abbreviation: Conc., concentration; KAE, Kaempferol.

To clarify the mechanisms of the suppressive effects of kaempferol on the IgE-dependent activation of MCs, we determined the expression levels of FcεRI on kaempferol-treated BMMCs. As shown in Figure 2A, the cell surface levels of FcεRI were slightly and significantly decreased by pretreatments for 24 h with 25 and 50 μM kaempferol, respectively, under the condition that c-kit expression levels were not affected. In addition, the mRNA levels of the FcεRI subunits were not decreased, but rather increased (particularly *Fcer1a* mRNA) in the kaempferol-treated BMMCs (Figure 2B). A time course analysis showed that a significant reduction in surface FcεRI level occurred at 2 h after the addition of kaempferol in the culture medium of BMMCs, and the reduction levels were time-dependently enhanced (Figure 2C). The treatment of BMMCs with cycloheximide, an inhibitor of protein synthesis, downregulated the cell surface levels of FcεRI and the cycloheximide-induced downregulation was significantly enhanced by kaempferol (Figure 2D). We also measured the FcεRI expression levels on cell surface and intracellular in the presence or absence of protein transporter inhibitors. Under the condition that the treatment with Brefeldin A and Monensin markedly increased the intracellular levels of FcεRI (Figure 2E right), the cell surface level of FcεRI, which was downregulated by the inhibition of protein trafficking, was significantly reduced by kaempferol treatment (Figure 2E left). In addition, we found that the intracellular level of FcεRI was also reduced by kaempferol. 

These results suggest that kaempferol suppressed cell surface expression of FcεRI in a transcription-independent manner and that the kaempferol-induced reduction in FcεRI expression was caused even when either protein synthesis or protein transporter was inhibited.

### 2.3. Kaempferol Suppressed LPS- and IL-33-Induced IL-6 Production without Affecting Cell Surface Levels of TLR4 and ST2 on BMMCs

**Figure 3:**
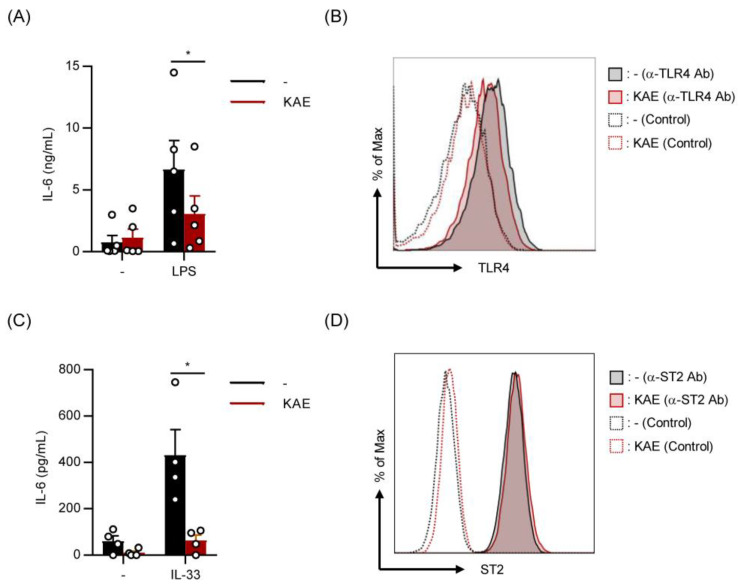
Kaempferol suppressed LPS- and IL-33-induced IL-6 production without affecting cell surface levels of TLR4 and ST2 on BMMCs. BMMCs were pre-incubated in the presence or absence of KAE (50 μM) for 24 h followed by each assay. (**A**) BMMCs were stimulated with LPS (1 μg/mL) for 3 h and the supernatant was collected to determine the concentration of IL-6. (**B**) The cell surface expression levels of TLR4 after KAE treatment. Typical histogram obtained in two independent experiments is shown. (**C**) BMMCs were stimulated with IL-33 (10 ng/mL) for 3 h and the supernatant was collected to determine the concentration of IL-6. (**D**) The cell surface expression levels of ST2 after KAE treatment. Typical histogram obtained in two independent experiments is shown. The data represent the mean ± SEM of four or five independent experiments, and two-tailed paired *t*-tests were used for statistical analyses (**A**,**C**). *, *p* < 0.05. Abbreviation: KAE, Kaempferol; α-TLR4 Ab, anti-TLR4 antibody; α-ST2 Ab, anti-ST2 antibody.

To further clarify whether kaempferol modulates the activation of BMMCs by other stimulation, we evaluated the effects of kaempferol treatment on cytokine production from BMMCs stimulated with LPS and IL-33. The treatment with kaempferol significantly suppressed LPS-induced IL-6 release from BMMCs (Figure 3A) without exhibiting apparent effects on the cell surface expression levels of TLR4 (Figure 3B). IL-33-induced IL-6 production was also significantly reduced in kaempferol-treated BMMCs (Figure 3C), without affecting IL-33 receptor levels in BMMCs (Figure 3D).

### 2.4. Kaempferol Treatment Activated NRF2 in BMMCs

The abovementioned results showed that the activation of BMMCs by LPS and IL-33 was significantly suppressed by the kaempferol treatment under the condition that the expression of their receptors was maintained. In the case of FcεRI, cell surface expression was significantly reduced in the presence of 50 μM kaempferol, which can be involved in the suppression of IgE-induced degranulation. However, the effect of 25 μM kaempferol on FcεRI expression was not striking, but the effect on the degranulation degree was significant. Thus, we demonstrated that kaempferol modulates intercellular events induced by various stimuli.

**Figure 4:**
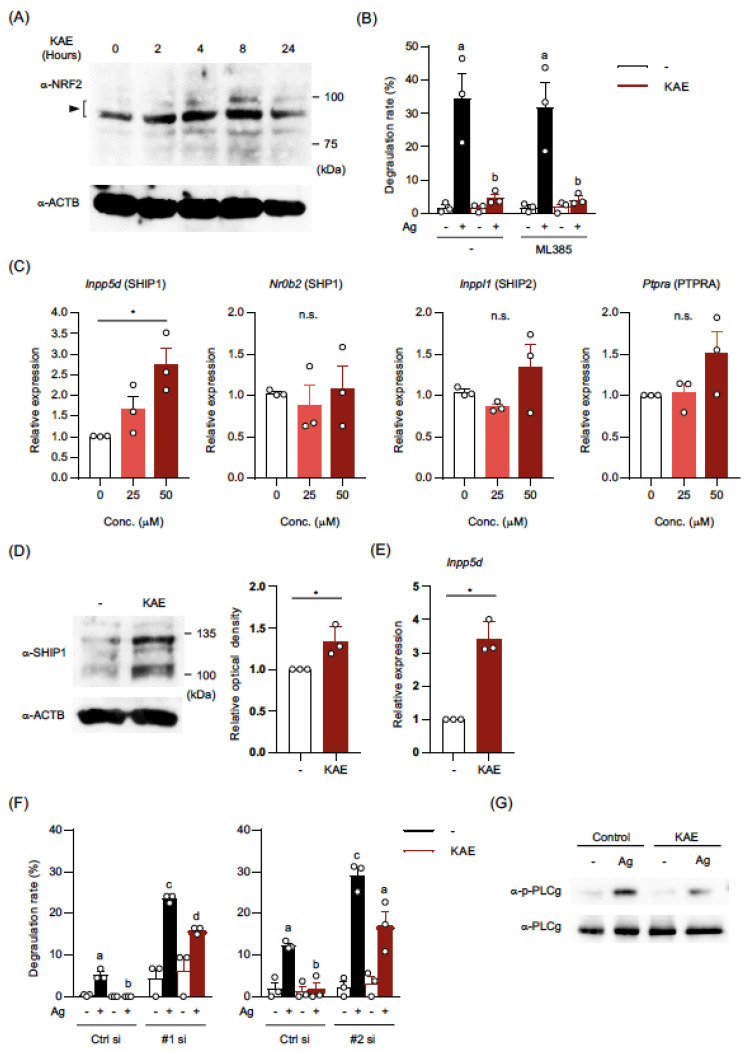
Effects of kaempferol on the NRF2 pathway and the expression of phosphatases in BMMCs. (**A**) Western blot analysis of NRF2. BMMCs were incubated in the presence of KAE (50 μM) and harvested at indicated time. Whole cell lysate containing 10 μg protein was added to each lane. The expected molecular weight of NRF2 was 97–100 kDa. (**B**) Effects of the NRF2 inhibitor on the suppression of IgE-mediated degranulation of BMMCs by KAE. BMMCs were incubated in the presence of the NRF2 inhibitor, ML385 (5 μM), and KAE (50 μM) for 24 h. Then, BMMCs were sensitized with anti-TNP-IgE and incubated in Tyrode’s buffer with or without TNP-BSA. The supernatant was collected for β-hexosaminidase assay. (**C**) mRNA expression levels of SHIP1 (*Inppd5*), SHP1 (*Nr0b2*), SHIP2 (*Inppl1*), and PTPRA (*Ptpra*). BMMCs pretreated with KAE for 24 h were harvested to assess mRNA levels by qPCR (normalized by β-actin). (**D**) Western blot analysis of SHIP1 protein. BMMCs treated with 50 μM KAE for 24 h were analyzed. Whole cell lysate containing 10 μg protein was added to each lane. Data of three independent experiments were quantified by calculating the band intensity, and shown as ratio to that of (anti-SHIP1)/(anti-β-actin) in control BMMCs. (**E**) mRNA expression levels of SHIP1 (*Inppd5*) in peritoneal MCs. Peritoneal MCs pretreated with KAE for 24 h were harvested to assess mRNA levels by qPCR (normalized by β-actin). (**F**) The effects of SHIP1 knockdown on the degranulation of BMMCs. BMMCs were transfected with SHIP1 siRNA (siRNA #1: left, #2: right) and cultured for 48 h followed by the treatment with KAE 10 μM for 24 h. After sensitization with anti-TNP-IgE, BMMCs were incubated in Tyrode’s buffer with or without TNP-BSA, and the supernatant was collected for β-hexosaminidase assay. (**G**) Western blot analysis of phosphorylated PLCγ and p-PLCγ. BMMCs were stimulated with IgE plus TNP-BSA for 10 min. Whole cell lysate containing 10 μg protein was added to each lane. The data shown in (**B**,**C**) (right), (**E**,**F**) represent the mean ± SEM of three independent experiments. The data shown in (**A**,**D**) (left), (**G**) represent a typical data of three other independent experiments. Tukey’s multiple comparison test (**B**,**F**), Dunnett’s multiple comparison test (**C**), and *t*-test ((**D**) right, and (**E**)) were used for statistical analyses. Means indicated by different lowercase letters are significantly different (**B**,**F**). *, *p* < 0.05; n.s., not significant. Abbreviation: Conc., concentration; KAE, Kaempferol.

The KEAP1-NRF2-HO-1 pathway is often activated by phytochemicals [16,17], and there are reports indicating that kaempferol activates the NRF2 pathway [18,19]. Several natural compounds, including resveratrol, exhibit anti-allergic effects by activating the NRF2 pathway in MCs [5,20,21,22]. Therefore, we examined the activation and role of NRF2 in kaempferol-treated BMMCs. A Western blot analysis showed that the amount of NRF2 protein was increased in BMMCs 2–8 h after the addition of kaempferol (Figure 4A), suggesting that kaempferol treatment activated the NRF2 pathway in MCs. However, the IgE-mediated degranulation was reduced in kaempferol-treated BMMCs even in the presence of ML385, an inhibitor of NRF2 (Figure 4B).

These results suggest that the NRF2 pathway was not involved in the suppressive effects of kaempferol on the activation of MCs, although NRF2 was activated by kaempferol treatment in MCs.

### 2.5. The Levels of mRNA and Protein in SHIP1 were Upregulated in Kaempferol-Treated BMMCs

Based on the observation that the inhibitory effect of kaempferol on A23187-induced degranulation was relatively moderate compared with that on IgE-mediated degranulation, intercellular events inhibited by kaempferol were likely upstream of Ca^2+^-signaling. Thus, we examined the effects of kaempferol on the expression of phosphatases, which inhibit the initiation of the FcεRI-mediated kinase cascade by dephosphorylating Tyr residues in ITAMs of FcεRI subunits and kinases associated with FcεRI. Among the candidate enzymes, SHIP1 was identified as a phosphatase whose mRNA level was increased in kaempferol-treated BMMCs (Figure 4C). By a Western blot analysis, we confirmed that the amount of SHIP1 protein was increased by kaempferol treatment in BMMCs (Figure 4D). To clarify whether the kaempferol-induced upregulation of SHIP1 expression was an observation limited in BMMCs, we examined the effect of kaempferol on SHIP1 expression in peritoneal MCs and revealed that the treatment of peritoneal MCs with 100 μM kaempferol for 24 h significantly increased mRNA level of SHIP1 without affecting cell viability (Figure 4E) and tended to suppress IgE-mediated activation (Supplemental Figure S1). This result indicates that the effect of kaempferol on SHIP1 upregulation was not restricted in BMMCs but also observed in MCs developed in biological hematopoietic environments. Furthermore, we evaluated the effects of kaempferol on the degranulation of BMMCs, in which *Inpp5d* siRNA was introduced, to investigate the role of increased SHIP1 in IgE-mediated degranulation. We confirmed that two different sequences of *Inpp5d* siRNAs significantly reduced the mRNA levels of *Inpp5d* in BMMCs (Supplemental Figure S2). These two knocked-down BMMCs exerted significantly enhanced degranulation compared to those of control siRNA transfected BMMCs (Figure 4F). Although kaempferol partially inhibited *Inpp5d* siRNA transfected BMMCs, it was still higher than that of control BMMCs without kaempferol treatment. We performed a Western blot analysis using specific Abs to examine the effects of elevated SHIP1 expression on IgE-mediated kinase activation in kaempferol-treated BMMCs. As shown in Figure 4G, we found that kaempferol treatment reduced the IgE-induced increase in phosphorylation of PLCγ. 

From these results, we conclude that kaempferol upregulated the expression of SHIP1 in MCs, which is involved in the suppressive effects of kaempferol on IgE-mediated activation of MCs.

## 3. Discussion

Several polyphenols have been reported to possess anti-allergic activities in vivo and suppress the activation of MCs [1,2,3,4,5]. Although a flavonoid kaempferol was also reported to inhibit allergic responses in vivo in mice by modulating MC function [7,8,9] or other cells [6], the molecular mechanisms underlying the inhibited activation of MCs remain unclarified. In the present study, we examined the effects of kaempferol on BMMCs, which were expected to have more natural phenotypes compared with cell lines, such as RBL-2H3 and LAD2, used in the previous study [7,8,9,10,11,12]. In our study, the kaempferol treatment inhibited the activation of BMMCs upon stimulation using IgE with Ag, LPS, and IL-33. Further analyses revealed that kaempferol downregulated the cell surface expression of FcεRI and upregulated the expression of SHIP1, which may be involved in the suppressive effects of kaempferol on MC activation.

In a previous study, kaempferol suppressed the surface expression of FcεRI on KU812F cells accompanied with reduced mRNA levels of FcεRIα and γ subunits [12]. Although the cell surface levels of FcεRI were downregulated in kaempferol-treated BMMCs in the present study as well, the mRNA levels of all of three subunits of FcεRI were not decreased by kaempferol in contrast to that of KU812F. Since the suppressive effects of kaempferol on FcεRI expression were still observed in the presence of inhibitors against protein synthesis or protein transporter, kaempferol appeared to have a reducing effect on FcεRI once expressed on the cell surface. In addition, we do not deny the suppressive effect of kaempferol on protein synthesis of FcεRI subunits even though the cell surface FcεRI was reduced by kaempferol in the presence of cycloheximide, because intracellular FcεRIα was decreased by kaempferol treatment. When FcεRIα, β, and γ subunits were transiently expressed in the HEK293T cell line in our preliminary experiment, surface expression of FcεRI on HEK293T cells was not reduced by the kaempferol treatment. From these results, we suggest that kaempferol downregulated the cell surface FcεRI mainly involving a post-translational event, which is caused in MCs but not in a human kidney cell line exogenously expressing FcεRI, and endocytosis may be causing the downregulation. Although several studies have shown that IgE-binding FcεRI is internalized in the cytoplasm from the membrane and is degraded, studies regarding endocytosis of empty FcεRI have not been reported. A ubiquitin ligase, CBL-B, is involved in the degradation of internalized IgE-binding FcεRI, resulting in the downregulation of cell surface FcεRI in MCs [23,24]. In our preliminary experiment, an increase in the mRNA level of CBL-B was observed in kaempferol-treated BMMCs, although the ubiquitination of FcεRI subunits was hardly detected. To clarify this issue, further analyses are required.

By a Western blot analysis, we found that IgE-induced phosphorylation of PLCγ was reduced in kaempferol-treated BMMCs. In previous studies, the activation of following signaling events was suppressed by kaempferol or its related flavonoids; the activation of PKCθ in human cord blood-derived MCs [13], Ca^2+^-influx in RBL-2H3 and HMC-1 [10], phosphorylation of cPLA2 and activation of Syk and PLCγ in RBL-2H3 [7], phosphorylation of ERK in RBL-2H3 [11], phosphorylation of PLCs, Lyn, Syk, ERK, p38 in RBL-2H3 [9], phosphorylation of Lyn, Syk, Btk, PLCγ in LAD-2 [8]. The above-mentioned suppression of signal transductions may reflect the downregulation of FcεRI, whose expression level affects the magnitude of IgE-induced activation [25], although the effects of flavonoids on FcεRI expression levels in these cells need to be confirmed.

In the present study, we found that kaempferol upregulated the expression of SHIP1 in MCs. The suppressive effect of kaempferol on IgE-induced degranulation was reduced in SHIP1 knocked-down BMMCs, suggesting the inhibitory role of SHIP1 in degranulation. In contrast, the knockdown of SHIP1 did not completely cancel the suppressive effect of kaempferol on degranulation. We speculate that the kaempferol-induced downregulation of FcεRI on cell surface may reduce the degranulation of SHIP1 knocked-down BMMCs. Alternatively, SHIP1 was slightly increased in kaempferol-treated BMMCs, even when *Inpp5d* siRNA was introduced, which may affect the degranulation degree. SHIP1 plays inhibitory roles in IgE-induced degranulation and cytokine production of MCs because SHIP1-deficient BMMCs exhibited hyper responses in the IgE-induced degranulation [26] and IgE-induced production of proinflammatory cytokines, including IL-6 [27]. Another study revealed that SHIP1 negatively regulates TLR4-mediated LPS responses in macrophages, in which knockdown and overexpression of SHIP1 were conducted [28]. In a study regarding LPS-induced proinflammatory cytokine production from BMMCs, the amount of TNF-α released from LPS-stimulated SHIP1 knockout (KO) BMMCs was higher than that of wild-type BMMCs [29]. Considering that MC-dependent allergic inflammation was exacerbated in SHIP1 KO mice accompanied by MC hyperplasia [30], an increase in SHIP1 in MCs may exhibit anti-allergic effects in vivo. We also found that kaempferol effectively inhibited IL-33-induced activation of BMMCs. Since IL-33R/ST2 is expressed in human MCs in contrast to TLR4, which is not detected in human MCs and mouse peritoneal MCs at steady state in a proteome analysis [31], the inhibitory effect of IL-33-mediated signaling may be more important than that on TLR4 signaling for applications of human diseases. Although we need to clarify the detailed mechanisms of the suppressive effects of kaempferol on IL-33-mediated activation of MCs, including the involvement of SHIP1 in this effect, kaempferol may be useful to prevent or treat IL-33-mediated allergic diseases such as asthma.

In the present study, we also observed the increase in NRF2 protein levels in kaempferol-treated BMMCs. A previous study reported that resveratrol, a well-known polyphenol, suppressed C48/80-induced pseudoallergic reactions in vivo and MRGPRX2-mediated MC activation in vitro by upregulating NRF2 expression [5]. In contrast, in our experimental conditions, the inhibition of NRF2 did not affect the IgE-induced degranulation and kaempferol-mediated suppression. Although the activation of the NRF2 pathway in MCs was observed in several studies, showing the anti-allergic effects of natural compounds [5,20,21,22,32], the roles of NRF2 in MCs may be more complicated. In our preliminary experiments, NRF2 deficiency did not enhance IgE-induced passive anaphylaxis in mice and IgE-induced degranulation of BMMCs, but rather reduced several MC-mediated responses in vivo and in vitro.

Kaempferol treatment upregulated the levels of mRNA and protein of SHIP1 in MCs, suggesting the effect of kaempferol on the transcription of the gene *Inpp5d*, encoding SHIP1. We will examine the roles of kaempferol on the transactivation of the *Inpp5d*. Natural compounds were often identified as ligands of nuclear receptors, and kaempferol was previously reported to function as a ligand of AhR [33]. However, in our preliminary experiments using siRNA, the involvement of AhR in the inhibitory effects of kaempferol on MCs was not observed.

In the present study using BMMCs, we found that kaempferol reduced the surface expression of FcεRI and increased the expression of SHIP1, which may be involved in the inhibitory effects of kaempferol on IgE-, LPS-, and IL-33-induced activation of MCs.

## 4. Materials and Methods

### 4.1. Mice and Cells

BMMCs were generated from BM cells of C57BL/6 mice (Japan SLC, Hamamatsu, Japan) by cultivation under IL-3-supplemented condition as previously described [34]. Peritoneal MCs were prepared from peritoneal exudate cells of mice as previously reported protocol with some modification [35]. Briefly, peritoneal cells were cultivated in the presence of SCF (10 ng/mL) and IL-3 (10 ng/mL) for 2 weeks at 37 °C in a humidified atmosphere in the presence of 5% CO_2_. More than 95% of nonadherent cells were identified to be MCs by positive staining with anti-FcεRIα (clone MAR-1, BioLegend, San Diego, CA, USA) and anti-CD117 (clone 2B8, BioLegened) in flow cytometry analysis. Independent experiments were performed on different days with different preparations of BMMCs generated from different mice. All experiments using mice were performed following the guidelines from the Institutional Review Board at Tokyo University of Science, and the present study was approved by the Animal Care and Use Committees at Tokyo University of Science: K22005, K21004, K20005, K19006.

### 4.2. Activation of MCs

A degranulation assay was performed as previously described [36,37]. Briefly, BMMCs sensitized with anti-TNP mouse IgE (clone IgE-3, BD Biosciences, San Jose, CA, USA) were stimulated with TNP-BSA (LSL-LG1117, Cosmo Bio, Tokyo, Japan) in Tyrode’s buffer, and the culture supernatant and cells were harvested to determine β-hexosaminidase activity.

To investigate the cytokine release, BMMCs were stimulated with IgE and TNP-BSA using a culture medium, instead of Tyrode’s buffer. BMMCs in culture media were also stimulated with LPS (#3024, Fujifilm Wako, Osaka, Japan) or IL-33 (#210-33, Peprotech, Rocky Hill, NJ, USA).

### 4.3. ELISA

The concentrations of IL-6, and TNF-α were measured using ELISA kits (ELISA MAX series, BioLegend), and of IL-13 was determined using the mouse IL-13 DuoSet ELISA kit (DY413, R&D systems, Minneapolis, MN, USA).

### 4.4. Western Blot Analysis

Western blot analyses using anti-phospho-tyrosine (clone 4G10, Merck, Darmstadt, Germany), anti-NRF2 (clone D1Z9C, Cell Signaling Technology, Danvers, MA, USA), anti-SHIP1 (clone V-19, Santa Cruz Biotechnology), anti-PLCγ1 (clone D9H10, Cell Signaling Technology), anti-phospho-PLCγ1 (Tyr783) (polyclonal, Cell Signaling Technology), and anti-β-actin (clone AC-15, Sigma-Aldrich, St. Louis, MO, USA) were performed as previously described [37]. Quantification of SHIP1 expression was carried out with Image J (NIH, Bethesda, MD, USA).

### 4.5. Flow Cytometry

Cell surface expression levels of FcεRI, c-kit, TLR4, and ST2 were determined by flow cytometric analyses using FITC-labeled anti-mouse FcεRIα, APC-labeled anti-mouse CD117, PE-labeled anti-mouse CD284 (TLR4) (clone SA15-21, BioLegend), and FITC-labeled anti-mouse T1/ST2 (clone DJ8, BioLegend), respectively. For intracellular staining, a fixation buffer (#420801, BioLegend) and an intracellular staining perm wash buffer (10X) (#421002, BioLegend) were used according to the manufacturer’s instructions with PE-labeled anti-mouse FcεRIα. Brefeldin A (#420601) and Monensin (#420701) were purchased from BioLegend. Data collected using a MACSQuant Analyzer (Miltenyi Biotec, Bergisch Glandbach, Germany) or a FACSLyric (Becton Dickinson, Franklin Lakes, NJ, USA) were evaluated with FlowJo software (Tomy Digital Biology, Tokyo, Japan).

### 4.6. Quantification of mRNA Using Real-Time PCR

The total RNA extracted from BMMCs using a ReliaPrep RNA Cell Miniprep System (Promega, Madison, WI, USA) was reverse-transcribed to cDNA with a ReverTra Ace qPCR RT Master Mix (TOYOBO, Osaka, Japan). Quantitative PCR was performed using a Step-One real-time PCR system (Applied Biosystems, Waltham, MA, USA) with THUNDERBIRD SYBR qPCR Mix (TOYOBO) and primers. The primers used for measuring the mRNA levels of *Fcer1a*, *Ms4a2* (encoding FcεRIβ), and *Fcer1g* were described previously [37]. To measure the mRNA levels of *Inpp5d* (SHIP1), *Inppl1* (SHIP2), *Nr0b2* (SHP1), and *Ptpra* (PTPa), the following primers were used: *Inpp5d* forward; 5′-tgcagtcaatatggaacatcaag-3′, *Inpp5d* reverse; 5′-gagacgaattgagatgtgactcc-3′, *Inppl1* forward; 5′-tcagggtcactcatatcaaggtt-3′, *Inppl1* reverse; 5′-cgagtagtcttctcattccctga-3′, *Nr0b2* forward; 5′-ccacctatcgatttgaaagactg-3′, *Nr0b2* reverse; 5′-cgtttacccgagtagcgtagtaa-3′, *Ptpra* forward; 5′-cccaatactggccagaccaa-3′, *Ptpra* reverse; 5′-cctcgacagacacacggacat-3′.

### 4.7. siRNA Transfection

A Neon transfection system (Invitrogen, Carlsbad, CA, USA) was used to introduce siRNA into BMMCs as previously described [36]. The following siRNAs were purchased from Invitrogen: siRNA against *Inpp5d* (MSS236924 termed #1 si, and MSS236925 termed #2 si) and control siRNA (Stealth RNAi Negative Universal Control, #462000 for MSS236924, and #452001 for MSS236925).

### 4.8. Statistical Analysis

One-way analysis of variance followed by Tukey’s multiple comparison test or Dunnett’s multiple comparison test was performed to compare three samples or more, and a two-tailed Student’s *t*-test was used to compare two samples.

## Data Availability

Not applicable.

## Supplementary Materials

The supporting information can be downloaded at: https://www.mdpi.com/article/10.3390/ijms24065997/s1.
