# Supplementary material for: Kaempferol Suppresses the Activation of Mast Cells by Modulating the Expression of FcεRI and SHIP1
Source: Int J Mol Sci. 2023 Mar 22;24(6):5997. doi: 10.3390/ijms24065997 (PMC10059252; doi:10.3390/ijms24065997)
Supplement: Supplementary file 1 [file ijms-24-05997-s001.zip › ijms-2304517-supplementary.pdf]

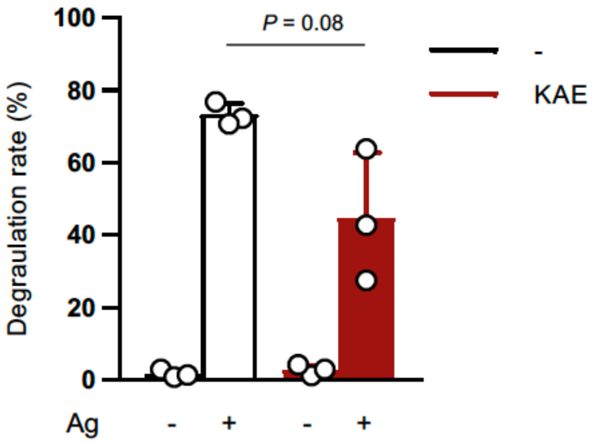

Supplemental Figure S1. Effects of kaempferol on degranulation of peritoneal mast cells. Peritoneal mast cells were pre-incubated in the presence or absence of KAE (100 μM) for 24 h. After sensitization with anti-TNP-IgE, the cells were incubated in Tyrode's buffer w/ or w/o TNP-BSA, and the supernatant was collected for β-hexosaminidase assay. The data represents the mean ± SEM of 3 individuals, and two tailed paired t-test were used for statistical analyses. Abbreviation: Ag, Antigen; KAE, Kaempferol.

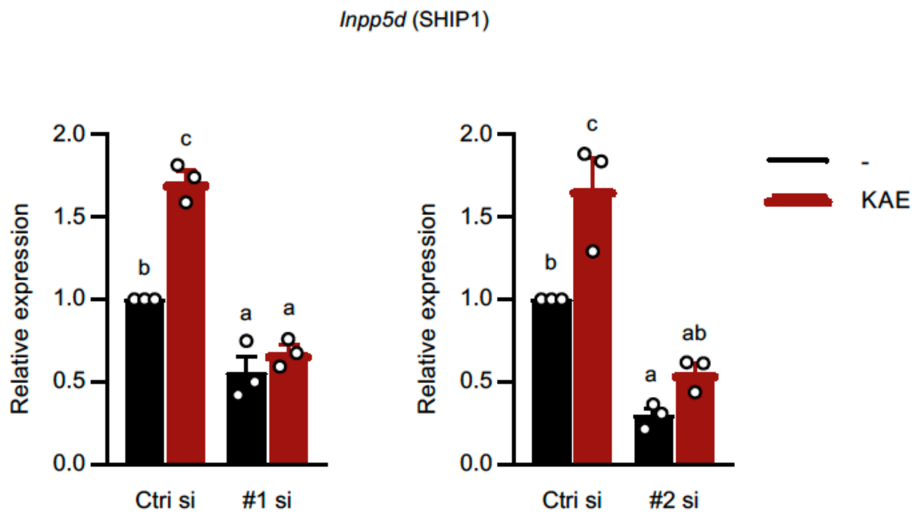

Supplemental Figure S2. The knockdown efficacy of SHIP1 by siRNA transfection. mRNA expression levels of SHIP1 (*Inppd5*) in siRNA transfected BMMCs. BMMCs were transfected with SHIP1 siRNA (siRNA #1: left, #2: right) and cultured for 48h followed by the treatment with KAE 10 μM for 24h. The cells were harvested to assess mRNA levels by qPCR (normalized by β-actin). The data represent the mean ± SEM of 3 independent experiments, and Tukey's multiple comparison test was used for statistical analyses. Abbreviation: KAE, Kaempferol.
